# GBA2-Encoded β-Glucosidase Activity Is Involved in the Inflammatory Response to *Pseudomonas aeruginosa*

**DOI:** 10.1371/journal.pone.0104763

**Published:** 2014-08-20

**Authors:** Nicoletta Loberto, Maela Tebon, Ilaria Lampronti, Nicola Marchetti, Massimo Aureli, Rosaria Bassi, Maria Grazia Giri, Valentino Bezzerri, Valentina Lovato, Cinzia Cantù, Silvia Munari, Seng H. Cheng, Alberto Cavazzini, Roberto Gambari, Sandro Sonnino, Giulio Cabrini, Maria Cristina Dechecchi

**Affiliations:** 1 Laboratory of Molecular Pathology, Department of Pathology and Diagnostics, University Hospital of Verona, Verona, Italy; 2 Department of Medical Biotechnology and Translational Medicine, University of Milano, Milano, Italy; 3 Department of Life Sciences and Biotechnology, Section of Biochemistry and Molecular Biology, University of Ferrara, Ferrara, Italy; 4 Department of Chemistry and Pharmaceutical Sciences, University of Ferrara, Ferrara, Italy; 5 Medical Physics Unit, Department of Pathology and Diagnostics, University Hospital of Verona, Verona, Italy; 6 Genzyme, a Sanofi Company, Framingham, Massachusetts, United States of America; University of Tübingen, Germany

## Abstract

Current anti-inflammatory strategies for the treatment of pulmonary disease in cystic fibrosis (CF) are limited; thus, there is continued interest in identifying additional molecular targets for therapeutic intervention. Given the emerging role of sphingolipids (SLs) in various respiratory disorders, including CF, drugs that selectively target the enzymes associated with SL metabolism are under development. Miglustat, a well-characterized iminosugar-based inhibitor of β-glucosidase 2 (GBA2), has shown promise in CF treatment because it reduces the inflammatory response to infection by *P. aeruginosa* and restores F508del-CFTR chloride channel activity. This study aimed to probe the molecular basis for the anti-inflammatory activity of miglustat by examining specifically the role of GBA2 following the infection of CF bronchial epithelial cells by *P. aeruginosa*. We also report the anti-inflammatory activity of another potent inhibitor of GBA2 activity, namely *N*-(5-adamantane-1-yl-methoxy)pentyl)-deoxynojirimycin (Genz-529648). In CF bronchial cells, inhibition of GBA2 by miglustat or Genz-529648 significantly reduced the induction of IL-8 mRNA levels and protein release following infection by *P. aeruginosa*. Hence, the present data demonstrate that the anti-inflammatory effects of miglustat and Genz-529648 are likely exerted through inhibition of GBA2.

## Introduction

Cystic fibrosis (CF) lung disease is characterized by progressive chronic infection and inflammation of the airways. The prolonged airway inflammation is an important aspect of the obstructive lung disease noted in CF patients. Resultant progressive remodeling leads to irreversible damage and fibrosis, which is a major cause of mortality in patients [1]. Significant efforts have been invested into developing therapies that address the underlying basis of CF. For example, recent efforts to identify small-molecule drugs that target a mutant CF transmembrane conductance regulator (CFTR) led to the successful development of a potentiator (Kalydeco) for patients who harbor the mutant G551D-CFTR [2]. Moreover, phase 3 clinical trials of Kalydeco in combination with the corrector lumacaftor for people with two copies of the F508del-CFTR mutation showed significant improvements in lung function and other key measures of the disease (http://www.cff.org/aboutCFFoundation/NewsEvents/2014NEWSArchive/6-24-VertexPhase-3-Results_Lumacaftor_Ibvacaftor.cfm). However, despite these very encouranging results, adjuvant therapies that abate the decline in pulmonary function in other patients are still needed. Examples include the potential deployment of new antibiotics, anti-mucolytic and anti-inflammatory drugs [3]. To date, the only non-steroidal anti-inflammatory agent that has been shown to be beneficial in CF patients is ibuprofen; however, its use can be associated with severe adverse effects, such as gastrointestinal bleeding [4]. Hence, the identification and development of novel and more potent anti-inflammatory drugs for CF airway disease remains a priority. The chemokine IL-8 is abundantly expressed at sites of chronic inflammation and appears to play a major role in driving the formation of neutrophil (PMN)-rich exudates in the lungs of CF patients [5]–[8]; thus its reduction is a key therapeutic goal in CF.

Sphingolipids (SLs) are a large group of lipids that are thought to modulate the pathophysiology of several respiratory disorders, including CF [9]–[11]. Ceramide, the central hub of SL metabolism, is generated by *de novo* synthesis or hydrolysis of complex SLs, such as sphingomyelin (SM) by acid sphingomyelinase (ASM) and glucosylceramide (GlcCer) by glucocerebrosidases [12]. Ceramide plays an important role in the infection by *P. aeruginosa* by reorganizing lipid rafts on cellular membranes into larger signaling platforms, which is a feature conducive to internalizing bacteria, inducing apoptosis and regulating the cytokine response [13]. Controversial findings on the association between abnormalities in SL metabolism and inflammation in CF have been reported. For example, ceramide has been identified as a key regulator of inflammation in CF airways in different CFTR-/- mouse models [14]. In contrast, decreased ceramide levels have been demonstrated in CFTR KO mice [15], and no significant difference has been found in basal ceramide levels in CFTR KO lung homogenates compared to wild type mice [16]. The possible explanation for this discrepancy appears to be the special diet required for the survival of CFTR KO mice, which severely affects the concentration of SLs [14]. Interestingly, an accumulation of ceramide, which has been directly correlated with neutrophilic lung inflammation, has been demonstrated in the lower airway of CF patients [17]. These findings suggest that the CF pathophysiology associated with infection by *P. aeruginosa* can be corrected, in part, by modulating ceramide levels to their normal physiological range, independent of the conflicting results obtained in different CF models. To date, there is some evidence that supports pharmacological interventions in SL metabolism as therapeutic agents for CF lung disease [14]–[21].

Given the emerging importance of SLs in respiratory disorders, novel drugs that selectively target different enzymes involved in SL metabolism are under development. Recently developed iminosugar-based inhibitors of GBA2 are of particular interest because of their good oral bioavailability and specific immune modulatory and chaperoning activities [22]. A well-characterized inhibitor is miglustat (*N*-butyldeoxynojirimycin, NB-DNJ), which is FDA-approved and EMA-designated for use in Europe and the USA for the treatment of type I Gaucher and other SL storage diseases. We previously demonstrated that miglustat exhibits an anti-inflammatory effect *in vitro* and *in vivo* by reducing *P. aeruginosa* induced immunoreactive ceramide levels [20], [23]. Moreover, miglustat can restore F508del-CFTR chloride channel activity in respiratory and pancreatic cells *in vitro* [24], [25] and in CF mice [26]. However, a recent clinical trial in CF patients did not provide evidence of efficacy, which may be related to the intra-individual variability of nasal potential difference (NPD) measurements or the short duration of exposure [27]. Nevertheless, a drug that is able to correct both the CF channel defect and reduce the inflammatory response is of interest and warrants further attention. Miglustat inhibits the enzyme ceramide glucosyl-transferase (GlcCerT), which catalyzes the first step in the glycosphingolipid biosynthetic pathway [28] with IC50 values in the low micromolar range. It also inhibits the activities of two different GlcCer degrading enzymes, glucocerebrosidase (GBA1) and the non-lysosomal β-glucosidase 2 (GBA2), with IC50 values in the high micromolar and nanomolar range, respectively [29], [30]. In addition, it is also a potent inhibitor of α-glucosidase [31]. Therefore, miglustat could affect the host response to *P. aeruginosa* through one or more of these SL metabolism pathways. The galactose analog of miglustat, *N*-butyldeoxygalactonojirimycin (NB-DGJ), also inhibits GlcCerT and GBA2, whereas its effect on GBA1 is less clear [30], [32], [33]. Similar to miglustat, NB-DGJ produces an anti-inflammatory effect in bronchial epithelial cells [25], which suggests a potential involvement of GlcCerT and/or GBA2 in the response of bronchial cells to *P. aeruginosa*. The non-lysosomal β-glucosidase GBA2, which is extremely sensitive to deoxynojirimycin-type inhibitors [34], is a membrane-associated enzyme located in the plasma membrane and ER of cells [29], [35]. GBA2 has been described as a single pass transmembrane protein with its catalytic domain facing the extracellular environment [29]. Because this enzyme can hydrolyze GlcCer directly at the cell surface, it might be involved in affecting transient local changes in bioactive SL concentrations.

To gain greater insights into the molecular basis of the anti-inflammatory activity of miglustat, we explored the potential involvement of GBA2 in the ceramide-mediated signaling processes following *P. aeruginosa* infection of CF bronchial epithelial cells. The effects of a potent inhibitor of GBA2, *N*-(5-adamantane-1-yl-methoxy)pentyl)- deoxynojirimycin or Genz-529648 as it is referred to in this report [4], [36], on the inflammatory response to *P. aeruginosa* were investigated and compared to miglustat and NB-DGJ. We also examined the impact of lowering the expression of GBA2 in human CF bronchial epithelial cells exposed to *P. aeruginosa* using siRNA oligonucleotides. The results obtained here demonstrate that GBA2 is a target of the anti-inflammatory effects of miglustat and Genz-529648. Thus, these compounds provide novel insights into the role of GBA2 in the signaling cascade activated by *P. aeruginosa* in CF bronchial epithelial cells.

## Methods

### Cell models

IB3-1 (LGC Promochem GmbH, Teddington, Middlesex, United Kingdom)[37] and CuFi-1 (a generous gift of A. Klingelhutz, P. Karp and J. Zabner, University of Iowa, Iowa City)[38] are human bronchial epithelial cells grown as previously described [24]. Primary airway epithelial cells, i.e., mainstem human bronchi, derived from CF individuals were obtained from “Servizio Colture Primarie” of the Italian Cystic Fibrosis Research Foundation and cultured as previously described [39].

### Bacterial strains

The reference *P. aeruginosa* strain, PAO1, was kindly provided by A. Prince (Columbia University, New York) and grown in trypticase soy broth (TSB) or agar (TSA) (Difco) as previously described [25]. Some experiments were conducted with organisms killed by heating to 65°C for 30 minutes.

### Inhibitors of SL metabolism

Miglustat and NB-DGJ were obtained from Toronto Research Chemicals, North York, ON, Canada. Genz-529648 was obtained from Genzyme, a Sanofi Company; amitriptyline was obtained from Sigma.

### Inflammatory response *in vitro*

Cells were treated with different inhibitors or solvent alone and then infected with PAO1 for 4 hours at 37°C as previously described [25]. The inflammatory response to PAO1 infection was studied at the transcriptional level by measuring IL-8 chemokine expression as previously described [20]. An enzyme-linked immunosorbent assay for the quantitative measurement of IL-8 protein release was performed using the Human IL-8 Instant ELISA kit (Bender MedSystems, Vienna, Austria).

### Cell toxicity

The effects of Genz-529648 on cell proliferation, viability and apoptosis were studied to evaluate the potential toxicity as detailed in the Supplement 3.

### GBA2 silencing

To perform silencing experiments of the GBA2 gene, a TriFECTa RNAi Kit (Integrated DNA technologies, Coralville, Iowa, IA) was used. Cells were transiently transfected with specific siRNA for GBA2 (sequence GGAUCAUGUUUGGAGCUA) or scrambled (CGUUAAUCGCGUAUAAUACGCGUAT) duplexes complexed with cationic liposomes Lipofectamine 2000 (Invitrogen, Carlsbad, CA) diluted in 1 ml serum-free cell culture medium. GBA2 siRNA or scrambled duplexes (10 nM) were added and incubated for 10 minutes. Liposome:duplex complexes (500 µL) were added to the cells grown in 2 cm^2^ wells and incubated at 37°C for 6 hours. The cells were washed twice with culture medium and maintained at 37°C for an additional 18 or 42 hours.

### Analysis of cell ceramide content

The analysis of cell ceramide content was performed via two different approaches: by the LC-MS and LC-MS/MS method [40] and by the metabolic labeling of cell SLs using (^3^H)sphingosine as a precursor. Both methods are detailed in the Supplement S1 and S2.

### Enzymatic activity

IB3-1 or CuFi-1 cells were treated with 2 µM miglustat, 10 nM Genz-529648 or solvent alone for 1 hour and the infected with heat-killed PAO1 for 4 hours. The cells were then scraped and centrifuged; the cellular pellets were resuspended in water containing protease inhibitors and sonicated. After protein determination, the β-glucosidase activities were assayed in the total cell lysates using the fluorigenic substrate 4-methylumbelliferyl-β-D-glucopyranoside (MUB-Glc) as previously described [41]. To discriminate between GBA1 and GBA2 β-glucosidase activity, the enzymatic assays were performed in the presence of 5 nM of Genz-529648 or 500 µM of Conduritol B Epoxide (CBE), respectively.

### Statistics

Results are expressed as the mean ± standard error of the mean. Comparisons between groups were made using Student’s t tests. Statistical significance was defined by p<0.05. In order to calculate the IC_50_ values, experimental data were fitted by nonlinear regression using the software “R Core Team, 2013, “R: A language and environment for statistical Computing”, R Foundation for Statistical Computing, Vienna, Austria, URL http://www.R-project.org.

## Results

### Genz-529648 reduces the expression of IL-8 in CF bronchial epithelial cells

**Figure 1:**
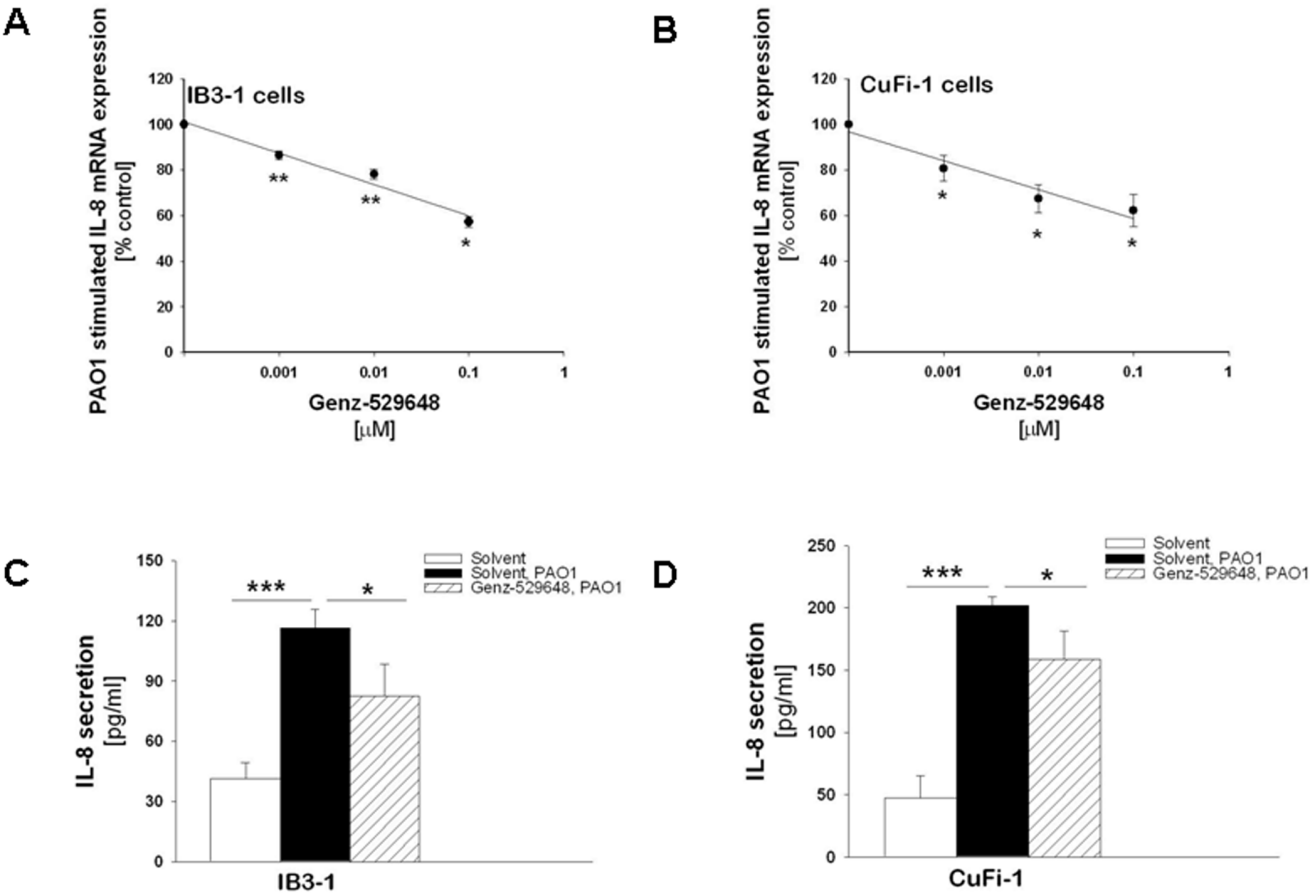
Genz-529648 reduces *P. aeruginosa* stimulated IL-8 mRNA expression and protein release. *Panels A and B. Genz-529648 reduces P. aeruginosa stimulated IL-8 mRNA expression.* IB3-1 (A) and CuFi-1 (B) cells were treated with a range of doses of Genz-529648 (1–100 nM) or solvent alone for 1 hour and then infected with PAO1 for 4 hours at 37°C. The inflammatory response was evaluated by studying the expression of IL-8 mRNA, which was measured by Real-time qPCR and obtained by comparing the ratio IL-8 and the housekeeping gene GAPDH between non-infected and infected cells. The results are expressed as the % of untreated cells and represent the mean ± standard error of the mean of 4 independent experiments in duplicate. Comparisons between groups were made by using Student’s *t* tests. *Panels C and D. Genz-529648 reduces the P. aeruginosa induced IL-8 secretion.* IB3-1 (C) and CuFi-1(D) cells were treated with Genz-529648 (100 nM) for 1 hour prior to infection with heat killed PAO1 for 24 hours. Data reported are the mean ± standard error of the mean of 4 independent experiments in duplicate. Comparisons between groups were made by using Student’s *t* tests.

Several hydrophobic deoxynojirimycin derivatives have been generated that can be used as research tools to probe the physiological relevance of GBA2. Complete inhibition of GBA2 can be realized in cells treated with low nanomolar concentrations of *N*-(5-adamantane-1-yl-methoxy)pentyl)- deoxynojirimycin (Genz-529648). GlcCerT and oligosaccharide chain-trimming glucosidases, which are sensitive to other hydrophobic deoxynojirimycin derivatives, are unaffected by Genz-529648 [34]. To determine a possible involvement of GBA2 in the inflammatory response to *P. aeruginosa* in bronchial epithelial cells, the effect of Genz-529648 was investigated and compared to miglustat and NB-DGJ. IB3-1 and CuFi-1 cells were treated with increasing amounts (1–100 nM) of the inhibitors for 1 hour prior to infection with *P. aeruginosa* (strain PAO1), and the IL-8 expression was then analyzed 4 hours post-infection. As shown in panels A and B in figure 1, Genz-529648 reduced the PAO1 induced increase in IL-8 mRNA levels by approximately 40% in both cell lines. These experiments were extended by measuring IL-8 chemokine secretion in the supernatants of IB3-1 and CuFi-1 cells. Thus, the cells were treated with Genz-529648 (100 nM) for 1 hour prior to infection with heat killed PAO1, and the supernatants were collected 24 hours later. Heat killed organisms were used to prevent bronchial cell death during the 24 hours of bacterial challenge. Figure 1, panels C and D, shows that Genz-529648 significantly decreased the amount of IL-8 released from the CF bronchial cells infected by PAO1 by approximately 30%, which is consistent with the results obtained at the transcriptional level (figure 1, panels A and B).

The effects of Genz-529648 in bronchial cells were then compared to miglustat and NB-DGJ, which also exhibit anti-inflammatory effects [25]. IB3-1 and CuFi-1 cells were treated with different concentrations of miglustat, NB-DGJ or Genz-529648 and infected with PAO1 as previously described; the IL-8 mRNA levels were then measured. As summarized in table 1, a similar maximal inhibition of approximately 50% was observed in both cell lines treated with miglustat, NB-DGJ or Genz-529648. However, the IC_50_ values of Genz-529648 in IB3-1 and CuFi-1 cells were considerably lower compared to miglustat or NB-DGJ, which indicated that it is a more potent inhibitor of the inflammatory response in CF bronchial cells. Moreover, the IC_50_ values of Genz-529648 at inhibiting IL-8 expression were of the same order of magnitude compared to that reported at inhibiting GBA2 [35], which suggests that the reduction in the inflammatory response to *P. aeruginosa* may have been mediated through its action on GBA2.

**Table 1:** Inhibition of *P. aeruginosa* stimulated IL-8 mRNA expression by alkylated iminosugars in IB3-1 and CuFi-1 cells.

IB3-1 cells				CuFi-1 cells				
*Inhibitor*	*IC_50_*	*CI*	*Maximal Inhibition*	*CI*	*IC_50_*	*CI*	*Maximal Inhibition*	*CI*
	*(μM)*	*(μM)*	*(%)*	*(%)*	*(μM)*	*(μM)*	*(%)*	*(%)*
**Miglustat**	2.2	1.4–3.4	51.6	51.1–53.3	1.98	1.4–2.7	51.5	51.0–57.3
**NB-DGJ**	0.27	0.16–0.44	45.0	39.0-52-0	0.39	0.004–3.8	53.0	41.0–55.0
**Genz-529648**	0.009	0.004–0.018	51.4	46.0–57.0	0.002	0.002–0.003	46.0	38.0–53.0

IB3-1 and CuFi-1 cells were treated with a range of doses of the alkylated iminosugars miglustat or NB-DGJ (0.5–100 µM) or Genz-529648 (1–100 nM) for 1 hour prior to infection with PAO1 (10–50 CFU/cell) for 4 hrs, and IL-8 mRNA expression was measured. IC_50_ values were calculated by fitting with a non-linear regression experimental data obtained in 4 different independent experiments performed in each cell line treated with each inhibitor.

IC_50_ values (*i.e.,* inhibitor concentration that results in 50% inhibition) were calculated by fitting experimental data with a non-linear regression according to the following formula: -log(*I*) =  *pKi* + log (V−v)/v.

*I* =  inhibitor concentration; v =  % inhibition; *pKi* =  IC_50;_ V =  maximal inhibition; CI = confidence interval 95%.

Although Genz-529648 is active at nanomolar concentrations, its potential toxicity on bronchial epithelial cells was investigated. To determine the impact on cell proliferation, IB3-1 cells were treated with increasing concentrations of Genz-529648 (from 0.001 to 1 µM), and the cell number/ml was analyzed after 4, 24, 48 and 72 hours. The results, which were derived from three independent experiments, indicate that the IC_50_ values calculated at these time points were always greater than 1 µM, which supports the concept that this compound is not cytotoxic at nanomolar concentrations and does not display inhibitory activity on CF bronchial cells. Cell viability, which was measured after 4 and 24 hours of treatment (figure S1), was always similar to the untreated cells and between 91.3 and 97.6%. At the same time points, treatment with Genz-529648 did not cause apoptotic effects, even when used at the 1 µM concentration (figures S2 and S3).

### Miglustat and Genz-529648 inhibit GBA2 activity in *P. aeruginosa* infected CF bronchial epithelial cells

**Figure 2:**
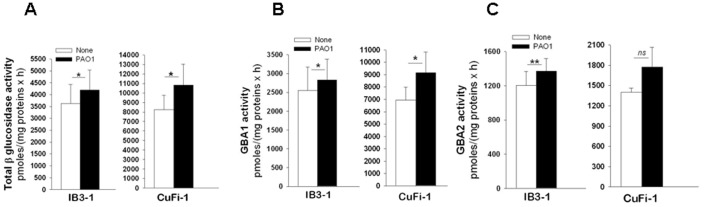
Infection with PAO1 increases β-glucosidase activity in IB3-1 and CuFi-1 cells. IB3-1 and CuFi-1 cells were infected with heat-killed PAO1 for 4 hours. The cells were then scraped and centrifuged; the cellular pellets were resuspended in water containing protease inhibitors and sonicated. Similar amounts of cellular proteins were used to perform the enzymatic assays to detect the activities of total β-glucosidase (A), GBA1 (B) and GBA2 (C), as reported in the Methods section. The data reported are the mean ± standard error of the mean of 4 (IB3-1) or 3 (CuFi-1) independent experiments in triplicate. Comparisons between groups were made by using Student’s *t* tests.

**Figure 3:**
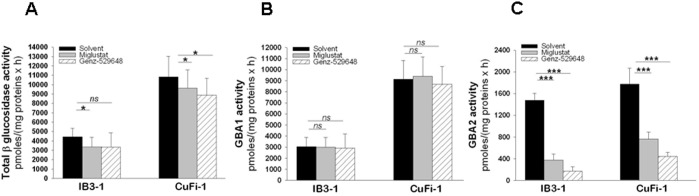
Miglustat and Genz-529648 inhibit GBA2 activity in IB3-1 and CuFi-1 cells infected by *P. aeruginosa*. IB3-1 and CuFi-1 cells were treated with [2 µM] miglustat, [10 nM] Genz-529648 or solvent alone for 1 hour prior to infection with heat-killed PAO1 for 4 hours. Total β–glucosidase (A), GBA1 (B) and GBA2 (C) activities were measured as indicated in figure 2. The data reported are the mean ± standard error of the mean of 3 (IB3-1) or 2 (CuFi-1) independent experiments in triplicate. Comparisons between groups were made by using Student’s *t* tests.

To ascertain the possible involvement of GBA2 in the signaling processes associated with *P. aeruginosa* infection, total β-glucosidase, GBA1 and GBA2 activities in the lysates of both IB3-1 and CuFi-1 cells infected by heat killed PAO1 were measured. To prevent potential interference because of bacterial glucosidase activities, the infected cells were subjected to washes with PBS that removed most bacteria; moreover, heat killed instead of living organisms were used. In addition, the residual GBA1 and GBA2 activities associated with heat killed bacteria were measured by enzymatic assays on the amounts of heat killed PAO1 from 20 to 30-fold higher compared to those used for the cell infection. The fluorescence associated with the PAO1 samples, which was the result of hydrolysis of the artificial substrate MUB-Glc, was less or the same extent of that identified in the negative controls, which indicates that heat killed PAO1 does not have detectable β-glucosidase activity. As shown in figure 2, a significant increase in total β-glucosidase (figure 2, panel A), GBA1 (figure 2, panel B) and GBA2 (figure 2, panel C) activities were observed in response to infection. The effects of pre-treatment with miglustat or Genz-529648 on β-glucosidase activity were then studied in both IB3-1 and CuFi-1 cells infected with PAO1. Total β-glucosidase was slightly reduced in both cell lines treated with the two inhibitors (figure 3, panel A), whereas GBA1 activity remained unchanged (figure 3, panel B). Importantly, both miglustat and Genz-529648 significantly decreased GBA2 activity in bronchial cells infected with *P. aeruginosa* (figure 3, panel C). These results demonstrate that miglustat and Genz-529648 inhibited the activity of GBA2 and support the hypothesis that GBA2 could be a target of the anti-inflammatory effects of deoxynojirimycin-type inhibitors.

### siRNA-mediated silencing of GBA2 in CF bronchial cells decreases IL-8 expression

**Figure 4:**
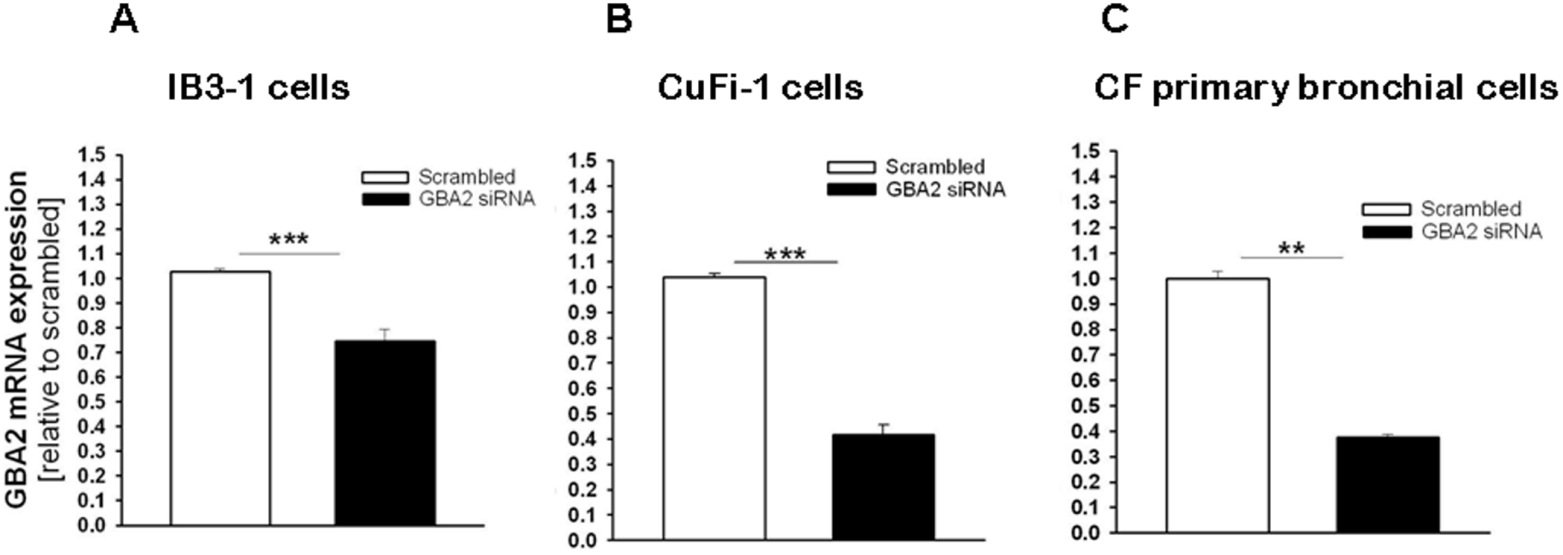
Transfection with GBA2 siRNA reduces the expression of GBA2 in CF bronchial cells. IB3-1 (A), CuFi-1 (B) or CF primary bronchial cells (C) were transfected with GBA2 siRNA or scrambled oligonucleotides for 24 h. GBA2 mRNA expression was measured by Real-time qPCR and obtained by comparing the ratio GBA2 and the housekeeping gene GAPDH between scrambled or siRNA treated cells. The data reported on the y-axis are relative to scrambled-treated cells and represent the mean ± SE of five (IB3-1, panel A), eight (CuFi-1, panel B) and four (CF primary bronchial, panel C) independent experiments performed in duplicate. Comparisons between groups were made by using Student’s *t* tests.

**Figure 5:**
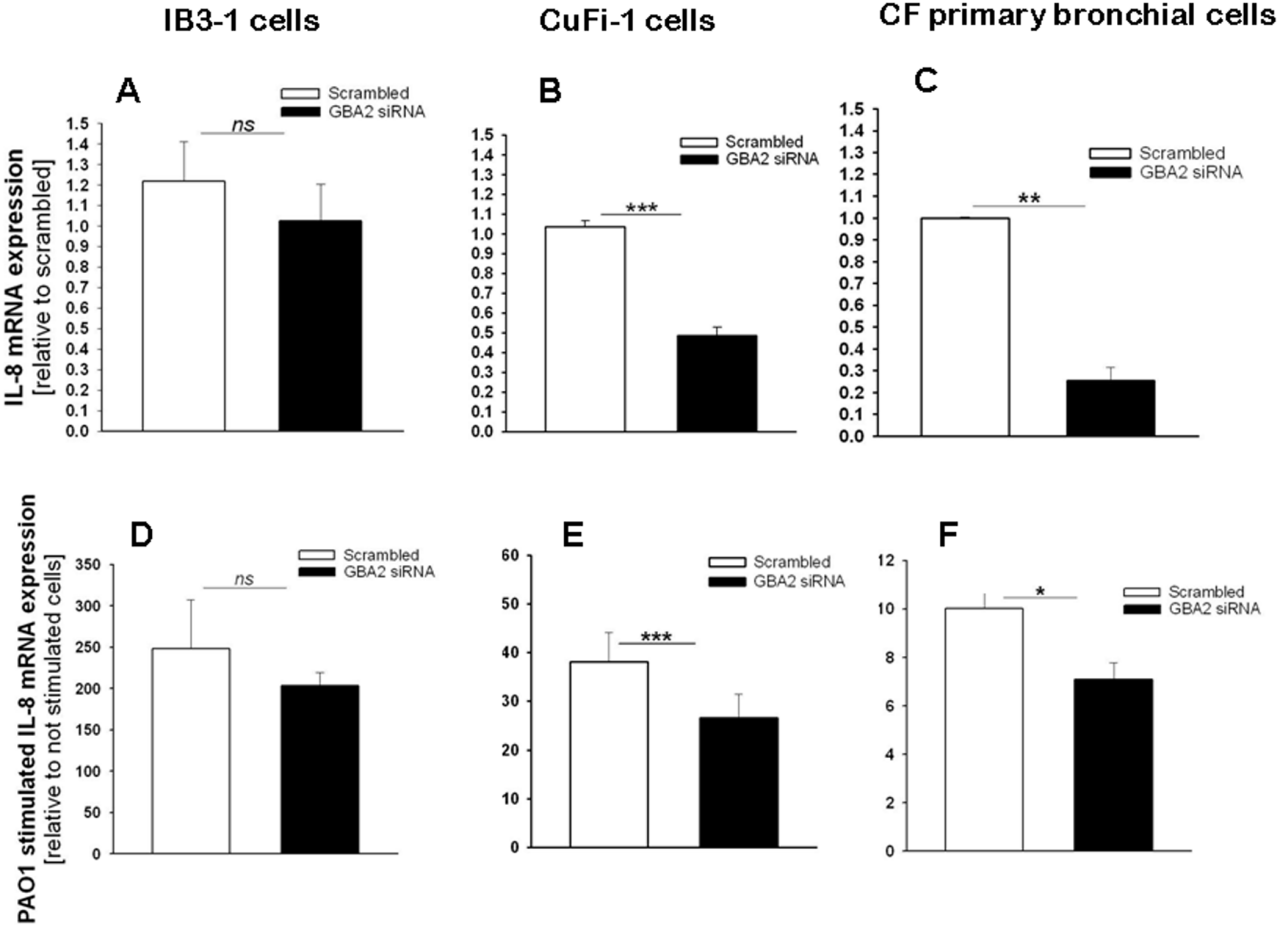
Reduction of IL-8 is associated with a relevant decrease of GBA2 expression in CF bronchial cells. IB3-1 (A), CuFi-1 (B) or CF primary bronchial cells (C) were transfected with GBA2 siRNA or scrambled oligonucleotides for 24 h and then infected with PAO1 (10–50 CFU/cell). IL-8 mRNA expression was measured as indicated in figure 1. The data reported on the y-axis are relative to scrambled-treated cells (A, B and C) or scrambled-treated uninfected cells (D, E and F) and represent the mean ± SE of five (IB3-1, panels A and D), eight (CuFi-1, panels B and E) and four (CF primary bronchial, panels C and F) independent experiments performed in duplicate. Comparisons between groups were made by using Student’s *t* tests.

**Figure 6:**
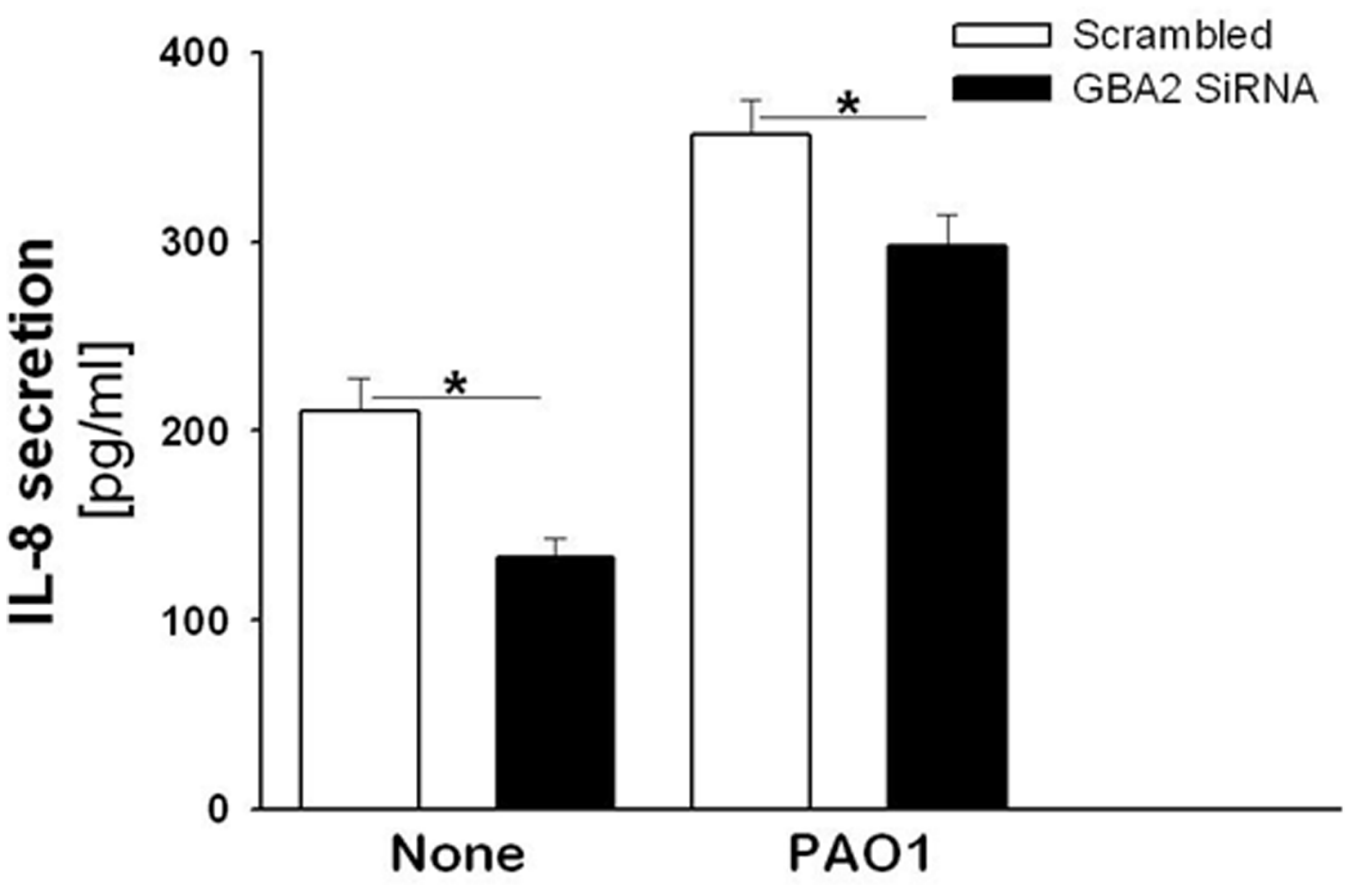
GBA2 silencing reduces the IL-8 protein release in CuFi-1 cells. CuFi-1 cells were transfected with GBA2 siRNA or scrambled oligonucleotides and then infected with PAO1 as indicated in figure 5. The supernatants were collected at the end of infection, and IL-8 protein release was measured as detailed in the “Methods” section. The data reported are the mean ± SE of eight independent experiments performed in duplicate. Comparisons between groups were made by using Student’s *t* tests.

**Figure 7:**
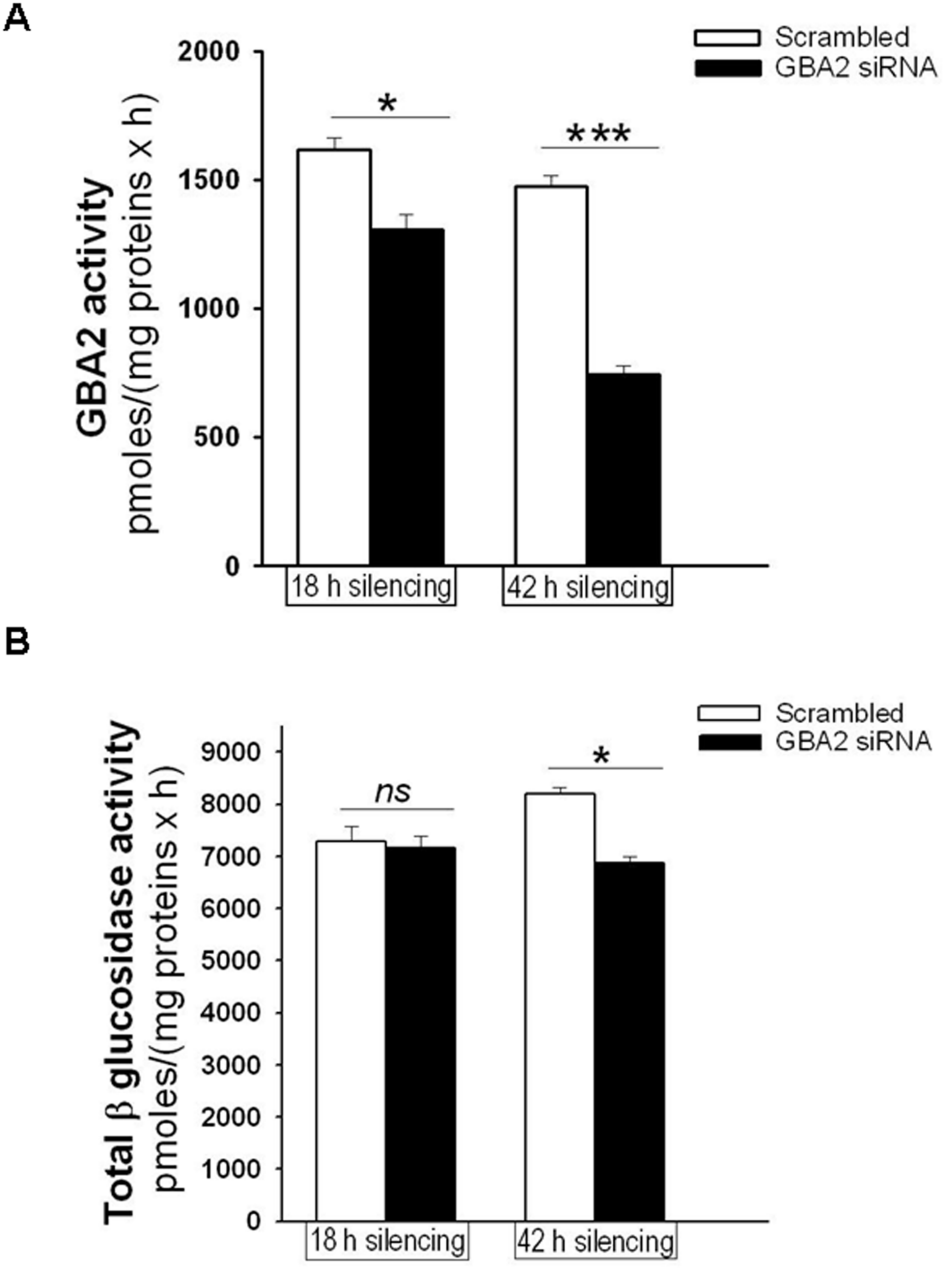
GBA2 silencing reduces GBA2 activity in CuFi-1 cells. CuFi-1 cells were transfected with GBA2 siRNA or scrambled oligonucleotides as indicated in figure 5. Eighteen or 42 hours after transfection, the cells were scraped and treated as indicated in figure 2. Total β-glucosidase (B) and GBA2 (A) activities were measured as reported in the Methods section. The data reported are the mean ± standard error of the mean of 2 independent experiments in triplicate. Comparisons between groups were made by using Student’s *t* tests.

To confirm if GBA2 is involved in the signaling cascade activated by *P. aeruginosa* infection of CF bronchial cells, the levels of IL-8 were measured following GBA2 silencing with siRNA oligonucleotides. The cells were transiently transfected with a siRNA that specifically targeted the degradation of human GBA2 mRNA or a control duplex scrambled siRNA. As shown in figure 4, panel A, transfection of IB3-1 cells with the GBA2-specific siRNA significantly reduced (30%) the level of expression of GBA2 mRNA. Transfection of CuFi-1 cells with the GBA2 siRNA produced a greater decrease (60%) in GBA2 mRNA levels (figure 4, panel B). The experiments were then repeated in primary CF bronchial cells, a cell model that closely resembles the native epithelium, where a decrease of GBA2 expression (∼60%) was also identified after transfection with the GBA2 specific siRNA (figure 4, panel C). As shown in figure 5, the silencing of GBA2 expression decreased IL-8 transcription in both uninfected cells (figure 5, panels A, B and C) and cells infected by *P. aeruginosa* (figure 5, panels D, E and F); however, in IB3-1 cells, the IL-8 reduction was not significant (figure 5, panels A and D). These findings were confirmed by measuring IL-8 protein levels in the supernatants of CuFi-1 cells at 4 hours post-infection with PAO1. As expected, the decrease in IL-8 mRNA expression was accompanied by a significant reduction in the secretion of IL-8 into the supernatant (figure 6). To provide evidence that the reduction of IL-8 levels is related to a decrease in GBA2 function, GBA2 activity was measured in GBA2 silenced CuFi-1 cells. Therefore, the cells were transiently transfected as previously described, and the total β-glucosidase and GBA2 activities in cell lysates were measured 18 and 42 hours after transfection. As shown in figure 7, panel A, GBA2 activity was significantly decreased at 18 hours after transfection. A further reduction of GBA2 activity resulted from measurements performed 42 hours after silencing. In these experimental conditions, transfection with siRNA that targeted GBA2 had an impact only on GBA2 activity, as demonstrated by the slight decrease of total β-glucosidase activity identified in GBA2 silenced cells (figure 7, panel B). These data, which demonstrate that lowering the expression and activity of GBA2 leads to a concomitant reduction in IL-8 levels, suggest a role for GBA2 in the inflammatory response induced by *P. aeruginosa* infection of CF bronchial cells.

### Increase of cell ceramide content induced by *P.* *aeruginosa* in CF bronchial cells

**Figure 8:**
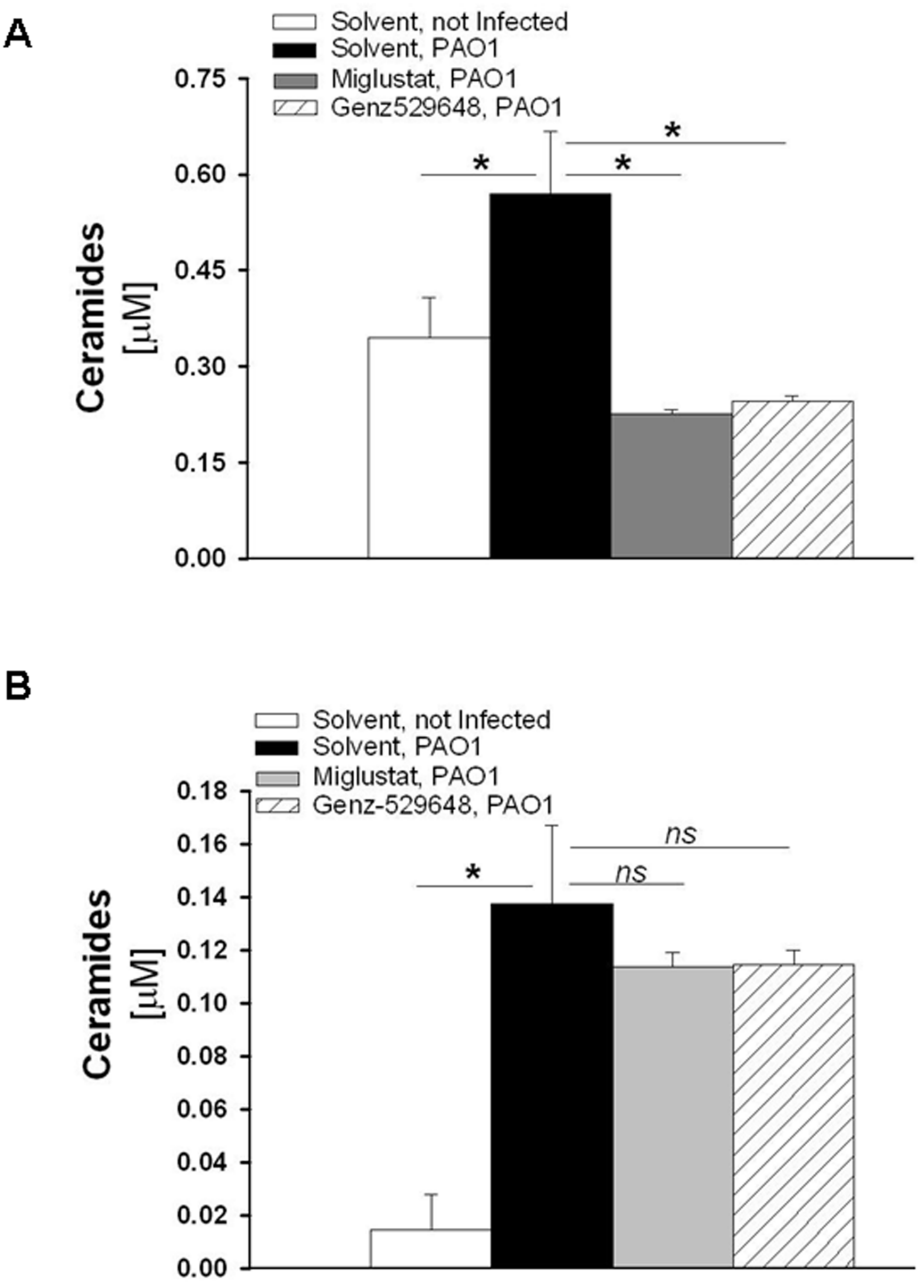
Infection with PAO1 increases whole cell ceramides in CF bronchial epithelial cells. IB3-1 (A) and CuFi-1 (B) cells were treated with [2 µM] miglustat, [10 nM] Genz-529648 or solvent alone and infected with PAO1 as indicated in figure 3. After infection, whole cell ceramides were analyzed by LC-MS and LC-MS/MS methods as described in the online supplement. The data reported are the mean ± SE of three independent experiments performed with both cell lines. Comparisons between groups were made by using Student’s *t* tests.

Inhibiting the catabolism of GlcCer by GBA2 could also lower ceramide levels and thereby reduce pulmonary inflammation in CF patients. We have previously shown that miglustat reduced the expression of immunoreactive ceramides (measured by immunofluorescence) induced by *P. aeruginosa* [20]. To assess the effect of *P. aeruginosa* infection on the total cell ceramide content, LC-MS and LC-MS/MS analyses were performed as detailed in the Supplement S1. In the PAO1 infected IB3-1 and CuFi-1 cells, a significant increase in ceramides was identified (figure 8, panels A and B), which indicated that the infection up-modulated whole cell ceramide levels. Treatment with miglustat or Genz-529648 significantly reduced whole cell ceramide in IB3-1 cells by approximately 50% (figure 8, panel A), whereas in CuFi-1 cells, a small, albeit not significant decrease in ceramide levels was identified (figure 8, panel B).

**Figure 9:**
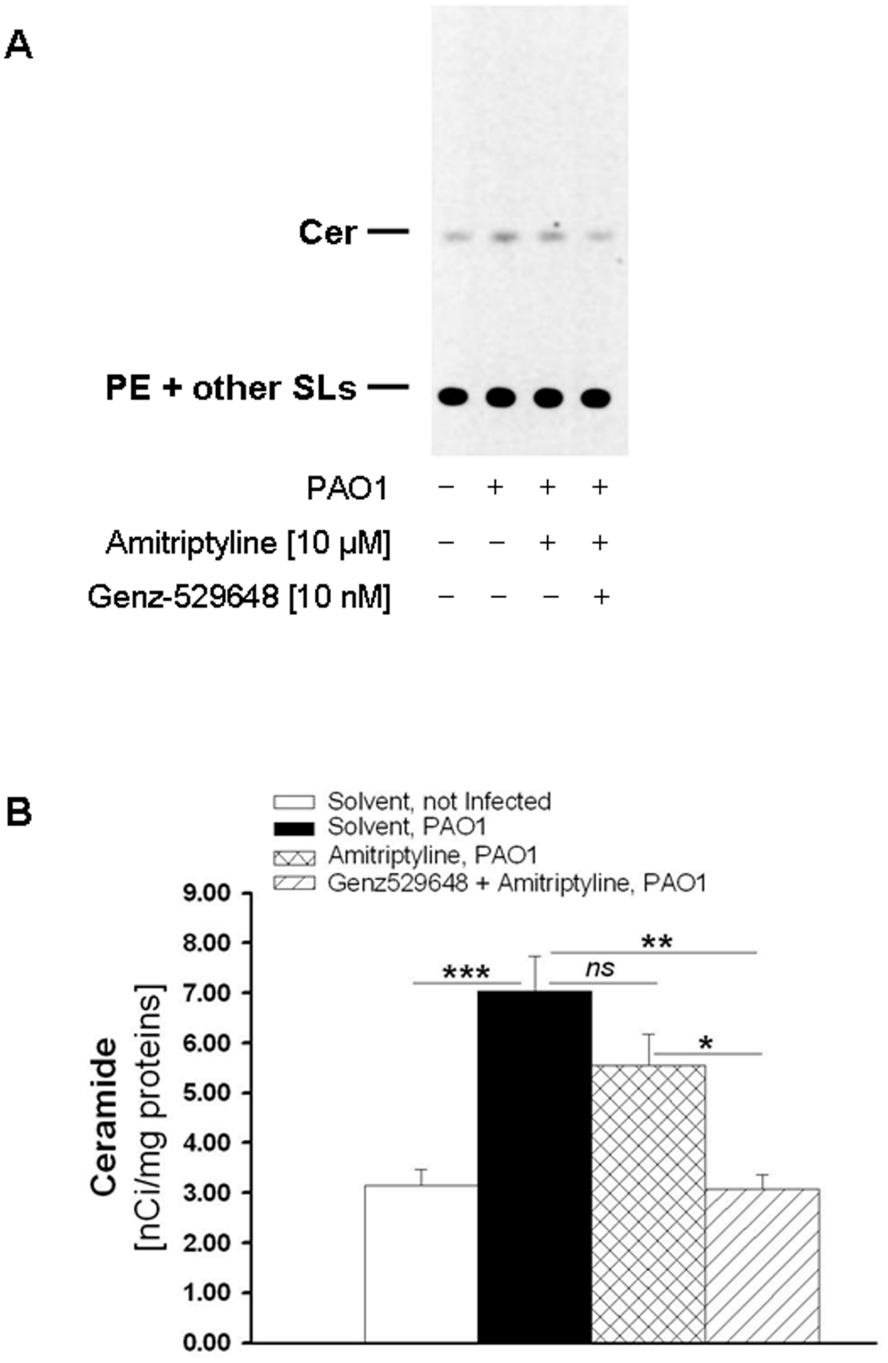
Treatment with Genz-529648 reduces the ceramide content in IB3-1 cells infected with PAO1. IB3-1 cells subjected to the SL metabolic labeling with (1-^3^H)sphingosine were treated with [10 µM] amitriptyline alone, in combination with [10 nM] Genz-529648, or with solvent alone and infected with PAO1 as indicated in figure 3. After lipid extraction, (^3^H)ceramide was separated from the other radioactive SLs by HPTLC, as detailed in the online supplement, and detected by digital autoradiography (total lipid extracts amounts corresponding to 4 µg of cellular proteins were applied on a 4-mm line. Time of acquisition: 48 hours). The digital autoradiography represents data obtained in three different experiments (A). The ceramide content was quantified by specific β-Vision software, and the data reported are the mean ± SE of three independent experiments. Comparisons between groups were made by using Student’s *t* tests (B).

To better evaluate the contribution of GSL catabolism to the ceramide increase following PAO1 infection, cell SL metabolic labeling was performed with the radioactive precursor sphingosine, which enables labeling of SLs at the steady-state. Thus, the effects of drug treatment on the radioactive ceramide content are only because of the modulation of the SL catabolism, thereby excluding the *de novo* pathway. To discriminate between the ceramide derived from SM catabolism and GSLs, we treated cells with amitriptyline alone, which is an inhibitor of ASM activity, or in combination with Genz-529648. IB3-1 and CuFi-1 cells were also subjected to the SL metabolic labeling with (1-^3^H) sphingosine and were then differently treated with 10 µM amitriptyline alone or in combination with 10 nM Genz-529648 and infected with PAO1 as detailed in the Supplement S2. The total lipid extracts (ELT) obtained from cells were subjected to HPTLC separation to distinguish ceramide from the other SLs. The radioactive ceramide was quantified by digital autoradiography (figure 9, panel A). In the CuFi-1 cells, the ceramide levels were under the sensitivity threshold of the digital autoradiograph used. By contrast, a significant increase in the ceramide content after PAO1 infection was observed in the IB3-1 cells. The treatment of cells with amitriptyline caused a slight reduction of ceramide, whereas a significant decrease of the ceramide content was observed when infected cells were treated with both amitriptyline and Genz-529648; these findings support a direct involvement of GBA2 in ceramide production (figure 9, panel B).

## Discussion

Recent advances in glycobiology have encouraged a search for novel drug molecules that address new biochemical targets. Iminosugars, which are carbohydrate-mimetics with a nitrogen atom replacing oxygen, have many attributes that make them suitable as small-molecule drug candidates. Pharmaceutical interest in these compounds is related to their ability to modulate carbohydrate processing, control cancer cell glycosylation, reduce viral and bacterial infectivity, alter immune responses and bind carbohydrate receptors [22]. The iminosugar miglustat, which was approved to treat type I Gaucher disease and Niemann-Pick type C disease, exerts an anti-inflammatory effect in CF human bronchial epithelial cells infected by *P. aeruginosa* and down modulates the neutrophil chemotaxis in murine lungs *in vivo* [20], [23]. Here, we report that the non-lysosomal β-glucosidase 2, is a target of the anti-inflammatory effects of miglustat and other deoxynojirimycin-type inhibitors used in this study. This contention is supported by the findings that: *i*) treatment of *P. aeruginosa* infected CF bronchial cells with Genz-529648, a potent inhibitor of GBA2, reduced the extent of inflammation; *ii*) the IC_50_ value of the anti-inflammatory effect of Genz-529648 was similar compared to the effect reported toward inhibiting GBA2 activity (33); *iii)* treatment of CF bronchial cells with miglustat or Genz-529648 inhibited GBA2; and *iv)* inhibition of GBA2 by siRNA lowered the expression of IL-8.

The alkylated iminosugars miglustat, NB-DGJ and Genz-529648 employed in this study inhibit GlcCerT, GBA1 and GBA2. However, the impact of these compounds on GlcCerT and GBA activities depends greatly on their dosage [30]. Lower concentrations of iminosugars primarily affect GBA2, whereas higher doses inhibit all enzymes. Notably, we obtained a reduction of *P. aeruginosa* stimulated IL-8 mRNA expression in CF bronchial cells treated with Genz-529648 at very low nanomolar concentrations, which completely inhibited GBA2 activity, but not GBA1 or GlcCerT [34]. Measurements of the sensitivity of GlcCerT, GBA1 and GBA2 to the inhibition by miglustat in different mammalian cells/tissues revealed IC_50_ values in the low µM, high µM and nM range, respectively. This finding indicates that GBA2 is more sensitive to miglustat compared to GlcCerT and GBA1 [30]. Although the IC_50_ values of miglustat at inhibiting *P. aeruginosa* stimulated IL-8 mRNA expression in CF bronchial cells (table 1) are higher compared to the IC_50_ values for inhibiting GBA2, they are substantially lower compared to GlcCerT and GBA1. Hence, GlcCerT and GBA1 are unlikely to have been the targets of the anti-inflammatory effects of miglustat. Furthermore, we previously reported that miglustat down modulates neutrophil chemotaxis *in vivo* at doses that are lower (100 mg/Kg) [20] compared to the doses necessary to affect GlcCerT (1800–2400 mg/Kg) [42], which further supports the notion that the primary effect of miglustat is on GBA2 activity.

GBA2 plays a role in extra-lysosomal GlcCer catabolism, producing ceramide that can then be rapidly converted into sphingomyelin [29]. Although the mechanism and function of extra-lysosomal GlcCer degradation are not well understood, GBA2 has recently been implicated in various pathologic conditions, such as neuronal diseases [43] or cancer [44], which supports a role of GlcCer in cell growth, proliferation and immunity. The present novel findings suggest that GBA2 may also be involved in modulating the inflammatory response to *P. aeruginosa* infection in CF bronchial epithelial cells. Indeed, total β-glucosidase, GBA1 and GBA2 activities were elevated in CF bronchial cells infected by *P. aeruginosa* (figure 2). As for the effects of infection on SLs, it should be noted that infection of host epithelial cells with *P. aeruginosa* activates host ASM levels, which leads to the generation of plasma membrane ceramide-enriched platforms that promote the internalization of bacteria, induce apoptosis and regulate the cytokine storm [13]. Based on the observations noted in our studies, it is possible that in addition to ASM, an overall activation of β-glucosidase activity may also be involved in the host cell response to infection. However, additional studies are needed to validate this assumption. Importantly, miglustat or Genz-529648, at the concentrations used in this study, strongly inhibited only GBA2 activity (figure 3); in parallel, we demonstrated a reduction of *P. aeruginosa* stimulated IL-8 mRNA expression and protein release in CF bronchial cells when GBA2 expression (figure 5) and function (figures 1 and 7) were decreased. These findings support the contention that GBA2 is involved in the inflammatory response to *P. aeruginosa.* GBA2 is typically associated with plasma- and/or ER-membranes in close proximity to the sites of GlcCer synthesis and ceramide conversion to SM [45]. Therefore, as GBA2 is in a key position for GlcCer-mediated signaling, it could be activated following interactions between *P. aeruginosa* and the host cell. It has been shown that GBA2 activation causes the phosphorylation of eukaryotic initiation factor 2α (eIF2α), and this event is associated with an increased expression of the ATF4 family of transcription factors [44]. Interestingly, phosphorylation of eIF2α has been observed in models of acute infection with *Clostridium difficile*, as part of the mucosal inflammatory response [46]. It can be speculated that GBA2 activation by *P. aeruginosa* leads to increased expression of the transcription factors that regulate the pro-inflammatory genes in CF bronchial cells.

**Figure 10:**
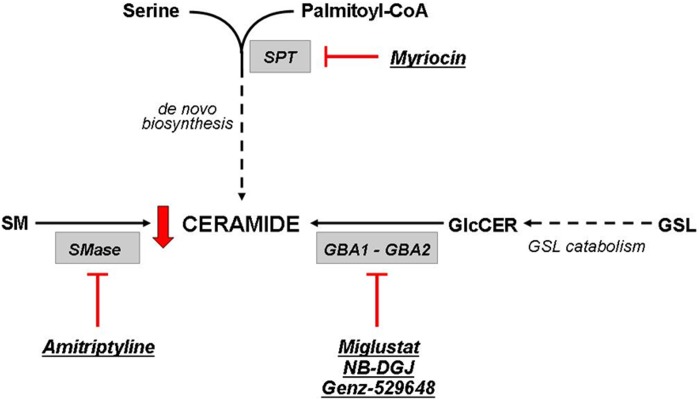
Metabolic pathways involved in ceramide formation. Schematic representation of the primary metabolic pathways involved in ceramide production. Ceramide can be produced by the *de novo* biosynthesis, the hydrolysis of sphingomyelin (SM) by the action of sphingomyelinases and the catabolism of glycosphingolipids (GSL). In particular, it has been observed that in CF bronchial epithelial cells, the use of inhibitors of these pathways resulted in a reduction of ceramide. Myriocin acts on the first step of the *de novo* biosynthesis through the inhibition of the Serine-palmitoyl transferase (SPT); amitriptyline inhibits the acid SMase (ASM) responsible for SM catabolism; and miglustat, NB-DGJ and Genz-529648 are inhibitors of the β-glucosidases GBA1 and GBA2, which are involved in the hydrolysis of the glucosylceramide (GlcCer).

The airway epithelium is known to play a key role in the initiation and regulation of inflammatory processes in response to pathogens. In addition to the classical cytokines and chemokines that are released by the respiratory epithelium, ceramide is another important factor in pulmonary host defense [11]. Here, we report an increase in whole cell ceramides in response to infection by *P. aeruginosa* (figures 8 and 9) in CF bronchial epithelial cells, which is consistent with the rise of ceramide levels at the plasma membrane previously described [20]. In CuFi-1 cells, which have a lower ceramide content compared to IB3-1 cells, we observed a slight decrease in ceramide levels by miglustat or Genz-529648 (figure 8, panel B). By contrast, IB3-1 cells infected with PAO1 and treated with both miglustat and Genz-529648 showed a marked decrease of ceramide content (figure 8, panel A). The increase in ceramide content following infection by PAO1 could be a result of different pathways, including the *de novo* biosynthesis [21], SM catabolism [13] and GSL degradation. When we evaluated the effects of drug treatment on the radioactive ceramide content that resulted only because of the modulation of SL catabolism, thus excluding the *de novo* pathway, we identified a marked increase in the cell ceramide content after PAO1 infection (figure 9). After ASM inhibition, we observed a slight decrease and a further, more significant ceramide reduction after the addition of the specific inhibitor of GBA2, Genz-529648 (figure 9), which strongly supports the direct involvement of GBA2 in the ceramide production after PAO1 infection of human bronchial epithelial cells. Thus, the information derived from the literature and the data presented here provide evidence that different inhibitors, such as miglustat and Genz-529648, amitriptyline [14] and myriocin [21], that target GBA2, ASM and ceramide *de novo* synthesis, respectively, could represent therapeutic tools to reduce ceramide levels and limit excessive lung inflammation in CF patients (figure 10). Nevertheless, drugs that target SL metabolism must be carefully titrated to normalize ceramide levels in CF airways, but not reduce ceramide concentrations below a critical level that would impair normal biological functions. Notably, systemic inhibition of ASM could negatively affect the host defense, which has been demonstrated by studies in mice that completely lack ASM and are unable to control infections [13]. Interestingly, no increased susceptibility to bacterial infections has been identified in patients affected by Gaucher disease, treated with miglustat [47] or in a mouse model of Sandhoff disease treated with Genz-529648 [48].

In summary, our study proposes GBA2 as a novel target to reduce the inflammatory response to *P. aeruginosa* in CF bronchial cells. These results further support the use of modulators of SL metabolism for CF lung inflammation. In addition, as GBA2 is sensitive to very low doses of miglustat, other alkylated iminosugars (NB-DGJ) and Genz-529648, our findings provide evidence to develop therapeutic options for CF lung inflammation using iminosugars, which can be effective at even low doses, thus limiting potential adverse effects.

## Supporting Information

Figure S1 **Viability profile of IB3-1 cells treated for 24 hours with the indicated concentrations of Genz-529648.**
(TIF)

Figure S2 **Apoptosis profile of IB3-1 cells treated for 24 hours with the indicated concentration of Genz-529648.**
(TIF)

Figure S3 **Apoptotic IB3-1 cells after 4 and 24 hours of treatment with the indicated concentrations of Genz-529648.**
(TIF)

Supplement S1 **Analysis of cell ceramide levels using LC-MS and LC-MS/MS.**
(DOC)

Supplement S2 **Analysis of cell ceramide levels by cell SLs labeling with (^3^H)sphingosine.**
(DOC)

Supplement S3 **Cellular toxicity of Genz-529648.**
(DOC)
