# Supplementary figures and images for: GBA2-Encoded β-Glucosidase Activity Is Involved in the Inflammatory Response to Pseudomonas aeruginosa
Source: PLoS One. 2014 Aug 20;9(8):e104763. doi: 10.1371/journal.pone.0104763 (PMC4139313; doi:10.1371/journal.pone.0104763)

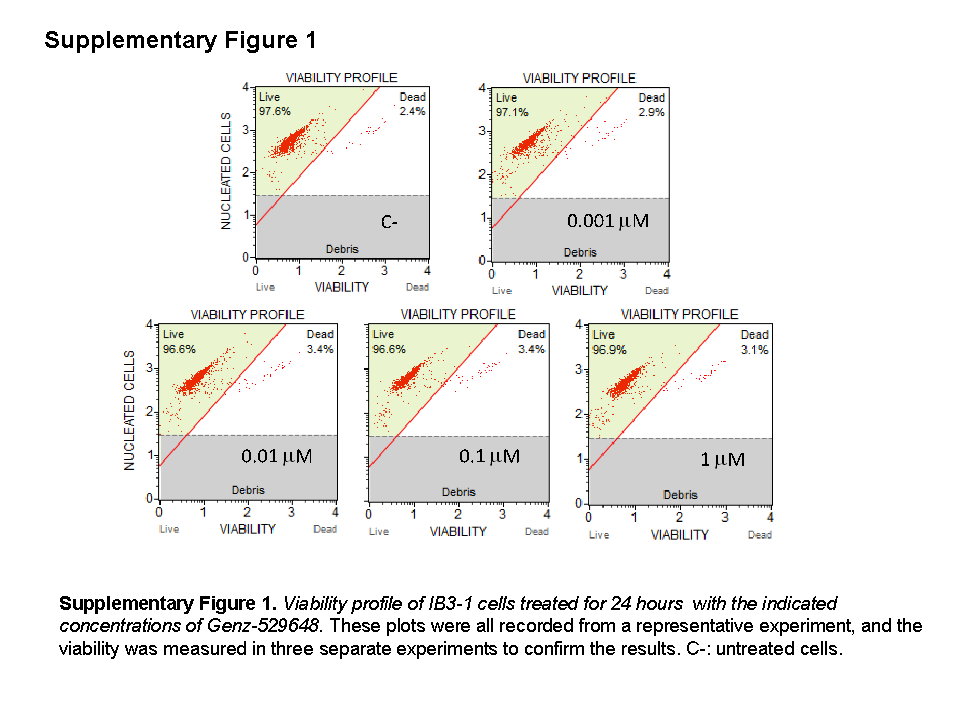

Supplement: Figure S1 — Viability profile of IB3-1 cells treated for 24 hours with the indicated concentrations of Genz-529648. (TIF) [file pone.0104763.s001.tif]

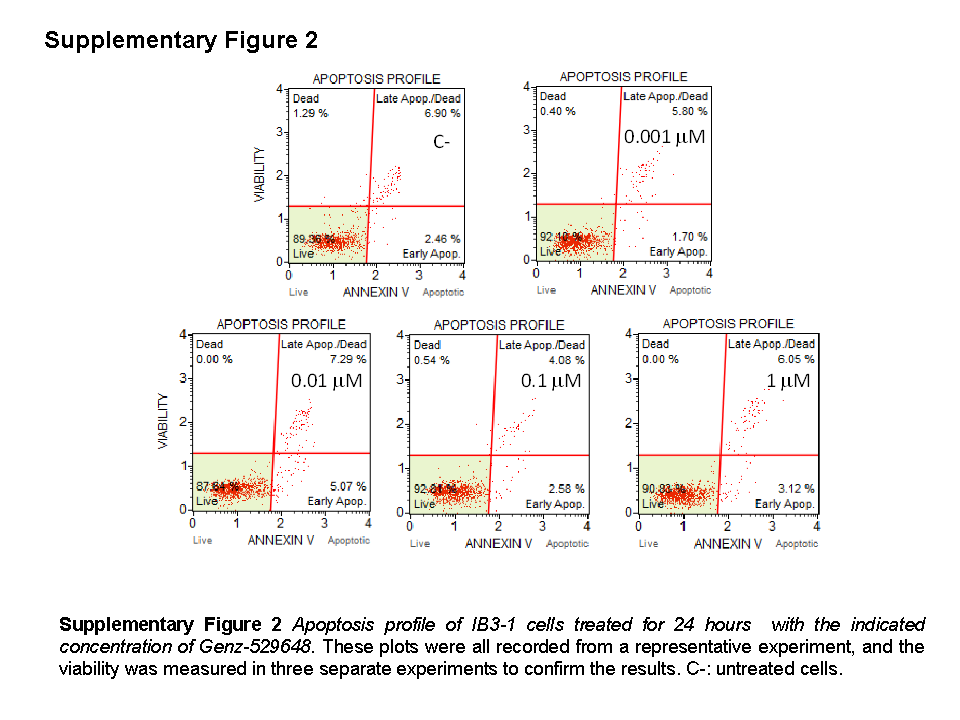

Supplement: Figure S2 — Apoptosis profile of IB3-1 cells treated for 24 hours with the indicated concentration of Genz-529648. (TIF) [file pone.0104763.s002.tif]
