# Supplementary material for: GBA2-Encoded β-Glucosidase Activity Is Involved in the Inflammatory Response to Pseudomonas aeruginosa
Source: PLoS One. 2014 Aug 20;9(8):e104763. doi: 10.1371/journal.pone.0104763 (PMC4139313; doi:10.1371/journal.pone.0104763)
Supplement: Supplement S1 — Analysis of cell ceramide levels using LC-MS and LC-MS/MS. (DOC) [file pone.0104763.s004.doc]

SUPPLEMENTARY METHODS

*S1. Analysis of cell ceramide levels using LC-MS and LC-MS/MS:*

Materials and instrumentation

Water, methanol (MeOH; LC/MS grade) and tetrahydrofuran (THF; HPLC grade) were purchased from Sigma-Aldrich (Milan, Italy). Synthetic standard ceramides (purity: 90%) were acquired from US Biological (Swampscott, MA, U.SA.).

A Kinetex C8 HPLC column was used (100 x 2.1 mm, 2.6 µm particles) (Phenomenex, Torrance, CA, U.S.A.). HPLC was carried out using a modular Surveyor system (Thermo Scientific, Waltham, MA, USA) equipped with a micro-quaternary pump (including the degasser), an autosampler and a column compartment (both of which were thermostated). The MS detector was an LTQ-XL (Thermo Scientific, Waltham, MA, U.S.A.) equipped with a linear ion trap mass analyzer.

HPLC/MS analysis

The employed mobile phase for LC/MS analysis comprised a mixture of water (0.1% v/v HCOOH):MeOH (0.1% v/v HCOOH):THF at a ratio of 15:40:45. HPLC runs were performed under isocratic conditions at a flow rate of 60 μL/min. The column was thermostated at 35°C. The typical retention times of determined ceramides are listed in Tab. 1.

The APCI interface was used as an ion source due to the low polarity of ceramides. The *m*/*z* values for each molecular ion and respective MS/MS fragments produced by collision-induced dissociation (CID) are listed in Tab. 1. Additional tandem mass transitions of MS/MS ions give the product ion 264.3 *m*/*z* for all ceramides; this value represents a valid qualifier fragment because these ions are not derived from other sphingosine metabolites. The value corresponds to the loss of an N-linked fatty acid moiety and two hydroxyl groups [1].

APCI conditions were as follows: positive ionization mode; spray, 10 µA (3.30 kV); vaporizer, 275°C; capillary, 16 V, 275°C; and tube lens, 125 V.

Quantitative analysis was performed using the internal standard (IS) calibration method. Ceramide C14 was used as the IS.

Cell treatment

Cells were treated and infected as described above. After infection, the cells were washed with sterile PBS, scraped, placed into Pyrex borosilicate tubes and stored overnight at -80°C until lipid extraction. This procedure was performed by isolating a 0.1 ml cell suspension (10^6^cells) and adding 0.5 ml of methanol, followed by 0.25 ml of chloroform and the internal standards C14, C16, C18, C20 and C22. After sonication and incubation at 48°C overnight, the insoluble precipitate was eliminated by filtration and the solvent evaporated under N_2_. Samples were then analyzed using the LC-MS and LC-MS/MS methods [2].

*Table S1.* ***Chromatographic retention times and* m*/*z *values of the parent and fragment ions for each determined ceramide.***

| **Ceramide** | **Retention time**  **(min)** | **Parent ion (*m*/*z*)** | **Relative collision energy (%)** | **Fragment (*m*/*z*)** |
| --- | --- | --- | --- | --- |
| C14 (IS) | 4.9 | 510.2 | 30 | 474.4 |
| C16 | 5.3 | 538.4 | 30 | 502.4 |
| C18 | 5.6 | 566.4 | 25 | 530.4 |
| C20 | 6.0 | 594.4 | 25 | 558.5 |
| C22 | 6.5 | 622.3 | 20 | 586.5 |

**REFERENCES**

1. Gu M, Kerwin JL, Watts JD, Aebersold R (1997) Ceramide Profiling of Complex Lipid Mixtures by Electrospray Ionization Mass Spectrometry. *Anal Biochem* 244: 347.
2. Sullards MC, Allegood JC, Kelly S, Wang E, Haynes CA et al (2007) Structure-specific, quantitative methods for analysis of sphingolipids by LC-tandem MS: “inside-out” sphingolipids. *Methods in Enzymology*: 432:83-115.
