# Supplementary material for: GBA2-Encoded β-Glucosidase Activity Is Involved in the Inflammatory Response to Pseudomonas aeruginosa
Source: PLoS One. 2014 Aug 20;9(8):e104763. doi: 10.1371/journal.pone.0104763 (PMC4139313; doi:10.1371/journal.pone.0104763)
Supplement: Supplement S2 — Analysis of cell ceramide levels by cell SLs labeling with (3H)sphingosine. (DOC) [file pone.0104763.s005.doc]

SUPPLEMENTARY METHODS

*S2. Analysis of cell ceramide levels by cell SLs labeling with (^3^H)sphingosine:*

Materials and instrumentation

(1-^3^H)sphingosine (radiochemical purity over 98%; specific radioactivity 0.86 Ci/mmole) was prepared as described [1].

High-performance silica gel precoated thin layer plates (TLC Kieselgel 60) were purchased from Merck. Methanol, chloroform, hexane, acetone and acetic acid were purchased from Sigma-Aldrich (Milan, Italy). (^3^H)ceramide, which was used as a chromatographic standard, was obtained through the extraction of SLs from cells fed (1-^3^H)sphingosine, purified and characterized as previously described [2].

SLs labeling

Cells were incubated at a preconfluent stage with 3×10^−8^ M (1-^3^H)sphingosine (3 ml/25 cm2 flask) for a 2 hours pulse followed by a 48 hours chase, dissolving the radioactive precursor of the SLs in the culture medium. After the pulse period, the medium was substituted with fresh medium without radioactive sphingosine during the chase period. This method allows the metabolic radiolabeling of all SLs, including ceramide (Cer), sphingomyelin (SM), neutral glycolipids, and gangliosides [3]. Tritium-labeled phosphatidylethanolamine (PE) was also obtained by recycling the radioactive ethanolamine formed during the catabolism of (1-^3^H)sphingosine.

Cell treatment

Forty-three hours after the end of the pulse period, cells were treated with 10 μM amitriptyline alone, with amitriptyline together with 10 nM Genz-529648, or with the solvent alone for 1 hour and then infected with heat-killed PAO1 for 4 hours. The cells were then scraped and centrifuged, and the cellular pellets were resuspended in water and lyophilized overnight. The lipids were then extracted with chloroform:methanol:water at a ratio of 2:1:0.1 by volume. The total lipid extract was analyzed using high-performance thin-layer chromatography (HPTLC) with the solvent system hexane:chloroform:acetone:acetic acid at a ratio of 20:70:20:4 by volume, followed by digital autoradiography performed with a Beta-Imager instrument (Biospace, Paris, France). The radioactivity associated with the ceramide was assayed using the specific β-Vision software provided by Biospace.

**REFERENCES**

1. Toyokuni T, Nisar M, Dean B, Hakomori S (1991) A facile and regiospecific tritiation of sphingosine: synthesis of (2S,3R,4E)-2-amino-4-octadecene-1,3-diol-1-3H. *J Labelled Compd Radio-pharm* 29: 567–574.
2. Tettamanti G, Bonali F, Marchesini S, and Zambotti V (1973) A new procedure for the extraction and purification of brain gangliosides. *Biochim Biophys Acta*. 296: 160–170.
3. Chigorno V, Palestini P, Sciannamblo MT, Dolo V, Pavan A et al (2000) Evidence that ganglioside domains are distinct from and caveolae in MDCK and human fibroblast cells in culture. *Eur J Biochem* 267: 4187–4197.
