# Supplementary material for: GBA2-Encoded β-Glucosidase Activity Is Involved in the Inflammatory Response to Pseudomonas aeruginosa
Source: PLoS One. 2014 Aug 20;9(8):e104763. doi: 10.1371/journal.pone.0104763 (PMC4139313; doi:10.1371/journal.pone.0104763)
Supplement: Supplement S3 — Cellular toxicity of Genz-529648. (DOC) [file pone.0104763.s006.doc]

SUPPLEMENTARY METHODS

*S3. Cellular toxicity of Genz-529648*

The effects of the compound Genz-529648 on cell proliferation, viability and apoptosis were studied to evaluate the potential toxicity in three independent experiments.

Proliferation assay

The possible antiproliferative effect of Genz-529648 was assessed on CF bronchial epithelial cells. IB3-1 cells were grown in LHC-8 medium supplemented with 5% FBS, as specified in the Methods section. Monolayers of 70% confluent cells were seeded onto 24 wells plates. After 24 hours, Genz-529648 was added at serial dilutions (0.001-1 μM), and the cells were incubated for 1 additional day. Cells were detached 4, 24, 48 and 72 hours after the addition, suspended in a physiological solution (50 μl of cells in 10 ml) and counted using a ZBI Coulter Counter (Counter Electronics, Hialeah, FL, U.S.A.).

Viability assay

The viability assay with increasing doses of Genz-529648 was performed on IB3-1 cells treated for 24 hours using the automated Muse method (Merck Millipore, Billerica, MA, U.S.A.). This procedure was carried out according to the manufacturer’s instructions, which include an in-house method of nuclear staining to assess cellular viability. Cells were harvested and diluted with a one-step addition of the mix-and-read Muse Count & Viability reagent (1:20), and following an incubation of 5 minutes at room temperature, the samples were analyzed. Data were acquired and recorded using the “*Count & Viability Software Module”* (Merck Millipore, Billerica, MA, U.S.A.).

Apoptosis assay

Annexin V and Dead Cell assays were performed on IB3-1 cells using the Muse method (Merck Millipore, Billerica, MA, U.S.A.). This procedure employs Annexin V to detect phosphatidyl serine (PS) on the external membrane of apoptotic cells. A dead cell marker was also examined as an indicator of cell membrane structural integrity. Cells were treated as described above, harvested and diluted with a one-step addition of the mix-and-read Muse Count & Viability reagent (1:2) and a one-step addition of the Muse Annexin V & Dead Cell reagent. After a 20 minutes incubation at room temperature, samples were analyzed with Triton X-100 0.01% as a positive control. Data were acquired and recorded using the “*Annexin V and Dead Cell Software Module”* (Merck Millipore, Billerica, MA, U.S.A.).
